# Supplementary material for: Persistent fatigue following SARS-CoV-2 infection is common and independent of severity of initial infection
Source: PLoS One. 2020 Nov 9;15(11):e0240784. doi: 10.1371/journal.pone.0240784 (PMC7652254; doi:10.1371/journal.pone.0240784)
Supplement: Attachment — Submitted filename: Response to Reviewers.docx [file pone.0240784.s001.docx]

Response to Reviewers

Dear Professor Madeddu and Editorial Board,

Many thanks for the very helpful feedback from both yourselves and the external reviewers. I have addressed each issue below, and have updated the manuscript accordingly.

**Editorial Comments**

1. Style requirement: formatting, including font size and capitalization, now reflect PLOS One’s style
2. Consent: clarification regarding written consent has been included
3. Race/ethnicity: the use of *Caucasian* has been replaced with *White*
4. Author number: the number of authors has been reduced in line with ICMJE criteria, with the addition of an acknowledgement section for those who contributed but do not meet authorship criteria

**Reviewer 1**

1. Stratification regarding timing of follow up has been provided and added to Table 1

**Reviewer 2**

*Introduction*

1. Additional information regarding the range of disease in acute infection has been added
2. Post-viral fatigue has been rephrased as post-infectious fatigue to reflect the presence of Q fever

*Methods*

3. Minor correction to statistical methods made to include median
4. The break-down range of physical and physiological fatigue is provided

*Results*

5. Deletion of (See Table 1)
6. Sentence referring to burden of healthcare worker infection has been moved to discussion
7. Rationale for controlling for age added to discussion, with reference; increasing age is known to be associated with increasing fatigue, hence it has been controlled for
8. Information regarding the use of targeted therapy (hydroxychloroquine and corticosteroids) added to results
9. Tables: Tables have been reformatted and percentages updated

*General Issues*

10. Article proofread and typos corrected
11. References checked and appropriate numbering and abbreviations added, in line with PLOS One’s requirements.

I hope we have satisfactorily addressed the issues raised.

Yours sincerely

Liam Townsend
