# Supplementary material for: Persistent fatigue following SARS-CoV-2 infection is common and independent of severity of initial infection
Source: PLoS One. 2020 Nov 9;15(11):e0240784. doi: 10.1371/journal.pone.0240784 (PMC7652254; doi:10.1371/journal.pone.0240784)
Supplement: Attachment — Submitted filename: Response to Reviewers.docx [file pone.0240784.s002.docx]

Response to Reviewers

Dear Professor Madeddu and Editorial Board,

Many thanks for the very helpful feedback. I have addressed each issue below, and have updated the manuscript accordingly.

**Editorial Comments**

N/A

**Reviewer 1**

N/A

**Reviewer 2**

1. The authors added that 41 patients received treatment with hydroxychloroquine. Have the authors checked if there are significant differences between the two groups (fatigued vs. non-fatigue)?.

Response: There was no difference in fatigue between those that received HCQ vs those that did not (17/35, 50% vs 18/36, 50%; *x*^2^ 6.67 p > 0.99). A line stating this has been added to the manuscript.

2. Have the authors verified if people admitted to the hospital have were related to chronic fatigue?

Response: No patients in the cohort, admitted or otherwise, had a pre-existing fatigue syndrome.

3. In table one, the difference in BMI between the two groups had a p-value slightly above the cut-off of significance. Have the authors verified if having an obesity condition was associated with the development of chronic fatigue? Different studies about chronic fatigue in people with cancer showed how overweight people and, in particular, obese people had an increased risk to have chronic fatigue (<https://doi.org/10.1007/s00520-019-04953-4>, <https://doi.org/10.1186/1471-2407-10-453>).

Response: the *p* value for BMI is 0.54, well above the cut-off for significance. There is no association between BMI and fatigue state (*p* 0.41 under unadjusted conditions).

4. In table one, the percentage should be rechecked. For example, the sum of the percentage in the row “Interval from COVID-19 Symptoms to Fatigue Assessment” about “people with fatigue” is 100.1%.

Response: Percentage for those attending at > 10 weeks corrected to 32.8%

5. In table one, in the row “Interval from COVID-19 Symptoms to Fatigue Assessment”, the interval seems wrong because ten days are missing (56-69; 79-83)

Rectified to 69 - 83

6. In the subtitle of table one, the abbreviation “CFQ” should be explained.

Response: legend amended

7. In the subtitle of table two, the abbreviation “CI” “CRP” and “IL” should be explained. The abbreviation “OR” is reported two times.

Response: legend amended

8. There are some typos; please check all the manuscript; for example, in the discussion, the authors wrote “These finding are independent of ageing effects” instead of “These findings are independent of ageing effects”; “Given that this cohort have no enduring evidence of active infection” instead of “Given that this cohort has no enduring evidence of active infection”.

Response: amended

9. The word “multidisciplinary” one times was written with the hyphen, one time without it.

Response: amended

10. In the introduction, the authors changed “post-viral fatigue” with “post-infection fatigue” as suggested. The sentence now is “Similarly, prominent post-infectious fatigue syndromes have been reported following Epstein-Barr Virus (EBV), Q-Fever and Ross River Virus (RRV) infections.” In my opinion, three important infectious disease are missing: HIV infection (<https://doi.org/10.1016/j.jpainsymman.2015.02.006>), HHV-6 infection (<https://doi.org/10.1155/1993/414602>), and rickettsiosis (<https://doi.org/10.4084/mjhid.2020.056>). I suggest adding them.

Response: Comments referring to these infections have been added.

I hope we have satisfactorily addressed the issues raised.

Yours sincerely

Liam Townsend
